# Selective defects of face familiarity associated to a left temporo-occipital lesion

**DOI:** 10.1007/s10072-020-04581-5

**Published:** 2020-07-10

**Authors:** Costanza Papagno, Edoardo Barvas, Marco Tettamanti, Guido Gainotti

**Affiliations:** 1 Center for Neurocognitive Rehabilitation (CeRiN) and Center for Mind/Brain Sciences (CIMeC), University of Trento, Via Matteo Del Ben, 5/b, 38068 Rovereto, TN Italy; 2 Department of Psychology, University of Milano-Bicocca, Milan, Italy; 3 Center for Mind/Brain Sciences (CIMeC), University of Trento, Rovereto, Italy; 4 Institute of Neurology, Catholic University of the Sacred Heart, Rome, Italy; 5 Laboratory of Clinical and Behavioural Neurology, IRCCS Santa Lucia Foundation, Rome, Italy

**Keywords:** Face processing, Familiarity feeling, Acquired prosopagnosia, Hemispheric specialization

## Abstract

Acquired prosopagnosia is usually a consequence of bilateral or right hemisphere lesions and is often associated with topographical disorientation and dyschromatopsia. Left temporo-occipital lesions sometimes result in a face recognition disorder but in a context of visual object agnosia with spared familiarity feelings for faces, usually in left-handers. We describe a patient with a left temporo-occipital hemorrhagic lesion unexpectedly resulting in a deficit of face familiarity, which could represent a mild form of associative prosopagnosia. Our patient failed to feel familiarity feelings even with very well-known famous faces but had neither visual object agnosia nor defects with semantics or naming of celebrities. This was confirmed even when the patient was re-tested a year later. We speculate that a graded lateralization of face processing could be at the basis of occasional cases of prosopagnosia.

**Electronic supplementary material:**

The online version of this article (10.1007/s10072-020-04581-5) contains supplementary material, which is available to authorized users.

## Introduction

Acquired prosopagnosia is a disorder of visual recognition specific to faces, associated with occipital or temporal bilateral lesions; occasionally, damage is restricted to the right hemisphere (RH) [1, 2], as reported in a review with 27 cases with neuroimaging plus four cases with surgical evidence of association between prosopagnosia and RH damage only [3]. An RH superiority in face processing [4] is confirmed by visual hemifield experiments [5–7], activation studies, e.g. [8, 9], EEG scalp topography, e.g. [10], TMS over the right occipital face area (OFA), e.g. [11], and intracranial stimulation, e.g. [12].

Despite this converging evidence, support to a left hemisphere (LH) contribution comes from neuroimaging and clinical findings. All the cited functional neuroimaging studies, though revealing that face perception results in a greater activation in right-sided face-processing network, show face-selective activation in the left fusiform region. Concerning clinical findings, there are four cases of prosopagnosia with LH lesions and intact RH [13–16]. Three of them [13, 14, 16] were left-handed. More patients (with less evidence of restricted LH lesions) have been described (see [17] for review). Some qualitative features allow distinguishing the rare instances of left temporo-occipital lesions causing face recognition defects from the more frequent cases of right homologous lesions. These features concerned (a) high proportion of left-handedness, (b) relative or complete sparing of familiarity feelings, and (c) coexistence of visual object agnosia. The explanation has been a defective ability to access both conceptual and person-specific semantic information from visual modality. The lack of familiarity found in right-brain-damaged patients contrasts with its preservation after left-brain-damaged patients [17]. The relations between loss of face familiarity feelings and disruption of RH structures have been confirmed [18], studying, in a large sample of neurodegenerative patients, the neuroanatomical substrates of three steps of famous face processing, namely, (a) familiarity judgment, (b) semantic/biographical information retrieval, and (c) naming. Familiarity correlated with right anterior middle temporal gyrus integrity, whereas performance in naming and semantic information retrieval significantly correlated with gray matter volume in the left anterior temporal lobe.

Most prosopagnosia cases are due to lesions of a bilateral network spanning from the inferior occipital gyrus, corresponding to the OFA [19], to the mid-fusiform gyrus, where the face fusiform area is (FFA; [20]), to the anterior temporal cortex (the AT of [21], or aIT of [22]). The inferior occipital areas mainly subsume the first stages of face perception [23], whereas a recognition-driven activity is carried out in FFA and aIT. Disconnection can cause a slightly different prosopagnosic picture [24], with intact perceptual face encoding and face memories. Probes of perceptual encoding generally involve match-to-sample or discrimination tasks.

Prosopagnosia can be associated with hemianopia, topographical skills impairment [25], word recognition deficits [26], achromatopsia [27], and visual agnosia (see [28]).

We studied a case of face familiarity loss with neuroimaging evidence of a left temporo-occipital lesion without the features typical of patients with face recognition defects from left temporo-occipital lesions [17]. Paradoxically, in this patient, only face familiarity feelings for famous people were selectively impaired, whereas semantic information retrieval and naming of people judged as familiar were intact. The selective impairment of mechanisms involved in familiarity was confirmed by the pathological score on face learning. A further interesting aspect was that, even if the lesion affected the OFA, which is regarded as involved in the fine-grained individual face analysis, he correctly matched unfamiliar faces. We thought, therefore, that a detailed report of this patient could be interesting, due to the variety of issues raised.

## Case report

A 56-year-old right-handed (with a left-handed brother) retired driver with 11 years of schooling came to our observation in May 2018 because of a right hemifield visual defect and calculation problems.

**Fig. 1:**
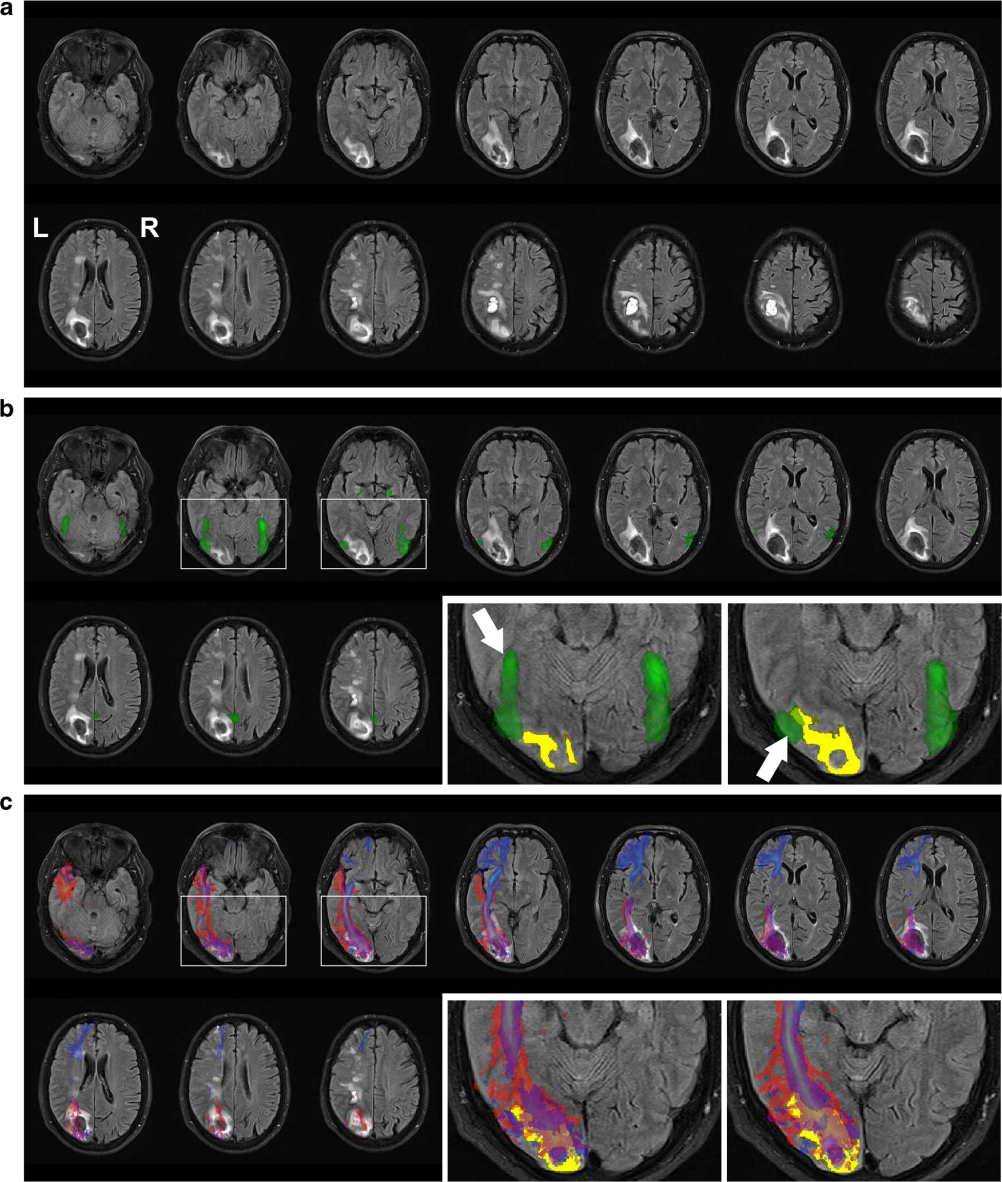
Patient’s brain MRI. **a** Fluid-attenuated inversion recovery (FLAIR) image of the patient’s brain, with transaxial slices (in neurological convention) revealing the presence of two left-hemispheric lesions, one centered around the inferior parietal lobule and the other one in the occipito-temporal territory. **b** Superimposed on the FLAIR image (green color) are the functional MRI probability maps of the “Atlas of Social Agent Perception” [44], representing the activation likelihood in a large cohort of the healthy population for the processing of face images. The maps have been warped to the patient’s native brain space. Rectangular areas (white outlines) of two adjacent transaxial slices are shown in greater magnification in the bottom right insets, with yellow indicating lesioned brain tissue automatically segmented by a lesion growth algorithm ([45]; initial threshold determined by visual inspection) as implemented in the LST toolbox version 2.0.15 (www.statisticalmodelling.de/lst.html) for SPM (www.fil.ion.ucl.ac.uk/spm). A downward white arrow (left inset) points to the approximate position of the fusiform face area (FFA), which is most likely not affected by the lesion. In turn, the occipital face area (OFA), indicated by an upward white arrow (right inset), is likely affected by the lesion. **c** Superimposed on the FLAIR image are the probabilistic tractography maps of the inferior longitudinal fasciculus (red color) and of the inferior fronto-occipital fasciculus (blue color) stemming from the “JHU White matter tractography atlas” [46]. The maps, representing the white matter tract probability in the healthy population, have been warped to the patient’s native brain space. As visible in the two rectangular (white outline) magnified insets, both white matter fascicles are most likely affected by the lesion (yellow color)

In February 2018, due to a left carotid aneurysm, the patient underwent an embolization procedure and was discharged with a triple anti-platelet therapy. He reported mild word finding difficulties for 3 days that spontaneously recovered. Ten days later, he suddenly claimed written language difficulties with spontaneous recovery. On March 4, the patient woke up with right homonymous hemianopia and was admitted to the emergency department of the local hospital, where the neurological examination revealed only hemianopia. A CT scan showed two intracerebral hemorrhages, a recent one in the left occipital lobe and a sub-acute (compatible with the reported written language and calculation difficulties) in the left parietal region. An MRI confirmed the two lesions (see Fig. 1).

The patient also complained impairment in recognizing people unless they spoke. Therefore, we investigated this ability in detail, after obtaining his written informed consent.The study was approved by the Ethical Committee of the University of Trento.

### General cognitive assessment

For all the tests used with this patient, normative data are available: raw scores are adjusted for age, for education, and, when indicated, for sex, according to the parameters estimated in a normal sample (200–321 neurologically unimpaired subjects) with a multiple regression model (see [29] for an extensive explanation of the standardization procedure).

On an extensive neuropsychological battery (see Table 1 and Supplementary Material) performed in our Cognitive Neurorehabilitation Center by a neuropsychologist, the patient showed no deficits except for mild difficulties with calculation. In particular, his performance was errorless in naming 48 objects pertaining to different living and non-living categories, ruling out also visual agnosia.

**Table 1:** General neuropsychological assessment

	Cut-off	Raw score	Adjusted score
Memory			
Digit span^(a)^			
Forward	< 4.26	6	6.04
Backward	< 2.65	4	4.10
Rey Auditory Verbal Learning Test^(b)^			
Immediate recall	< 28.53	40/75	40.7
Delayed recall	< 4.69	10/15	10.2
Modified Taylor Complex Figure-Delayed Recall^(c)^	< 8.40	21/36	19.8
Attention and executive functions			
Multiple Features Target Cancelation^(d)^			
Hits		11/13	11
Errors	> 2.77	0	0
Execution time (sec.)	> 135.73	116	109.97
Accuracy	< 0.869	0.923/1	
Frontal Assessment Battery^(e)^	< 13.48	17/18	17.1
Weigl’s Sorting Test^(f)^	< 8.1	12/15	13.3
Verbal fluency on phonological cue^(g)^	< 17.77	37	42.91
Language, calculation, and praxis			
Picture naming—nouns^(h)^	< 41.49	48/48	48
Picture naming—verbs^(i)^	< 36.87	50/50	50
Aphasia neuropsychological evaluation (ENPA)^(j)^			
Reading—words	< 6.4	10/10	10
Reading—non-words	< 4	5/5	5
Reading—sentences	< 1.3	2/2	2
Writing—words	< 6.3	9/10	8.4
Writing—non-words	< 1.4	4/5	3.3
Writing—sentences	< 0.6	2/2	2
Calculation—addition	< 2.2	3/3	3
Calculation—subtraction	< 1	1/3	0.8*
Calculation—multiplication	< 1.4	4/4	4
Ideo-motor apraxia^(k)^	< 28	36/36	
Body representation disorders			
Right-left orientation^(l)^	< 17	20/20	20
Finger agnosia^(l)^	< 48	60/60	60
Visuo-perceptual, visuo-spatial, and visuo-constructive abilities			
Ishihara Test—14-Plates^(m)^	< 10	14/14	14
Farnsworth-Munsell 100 Hue Color Vision Test (Errors)^(n)^	> 70	50	50
Screening for color defects^(o)^			
Color naming	< 24	30/30	30
Color recognition (pointing)	< 26	30/30	30
Memory for objects’ prototypical color	> 21	30/30	39
Modified Taylor Complex Figure—Copy^(c)^	< 28.87	34/36	33.7
Street’s Gestalt Completion Test^(p)^	< 2	10/14	9
Line orientation judgment^(l)^	< 19	29/30	30
Topographical orientation and topographical memory			
Topographical orientation test (Map of Italy)^(p)^	< 7.50	15/15	15
Topographical orientation test (Map of Trento) (qualitative)		5/5	5
Recognition Memory Test—Buildings^(q)^	< 21.41	26/30	26.14
Topographical Memory Test—Buildings (qualitative)		17/22	17
Topographical Memory Test—Trento (qualitative)		4/4	4

*Pathological scores

Raw scores are adjusted for age, for education, and, when indicated, for sex, according to the parameters estimated in a normal large sample with a multiple regression model. Adjusted scores < 5% one-sided non-parametric tolerance limit (with 95% CI) are considered pathological: inferential cut-off scores are therefore those at which or below which the probability that an individual belongs to the normal population is < 0.05

References for the neuropsychological tests

^(a)^Monaco et al., (2013) *Neurological Sciences, 34*(5), 749–754

^(b)^Carlesimo et al., (1995) *Archivio di Psicologia, Neurologia e Psichiatria, 56*(4), 471–488

^(c)^Casarotti et al., (2014). *Journal of Neuropsychology, 8*(2), 186–198

^(d)^Marra et al., (2013) *Neurological Sciences, 34*(2), 173–180

^(e)^Apollonio et al. (2005) *Neurological Sciences, 26*(2), 108–116

^(f)^Laiacona et al., (2000) *Neurological Sciences, 21*(5), 279–291

^(g)^Costa et al., (2014) *Neurological Sciences, 35*(3), 365–372

^(h)^Catricalà et al., (2013) *Neurological Sciences, 34*(6), 985–993

^(i)^Papagno et al., (2020) *Neurological Sciences* doi:10.1007/s10072-020-04353-1

^(j)^Capasso, R. & Miceli, G. (2001) Milan, Italy, Springer-Verlag

^(k)^De Renzi, et al., (1980) *Archives of Neurology, 37*(1), 6–10

^(l)^Ferracuti et al., (2000) Florence, Italy, Giunti Organizzazioni Speciali

^(m)^Ishihara, (2006) Tokyo, Japan: Kanehara Trading

^(n)^Farnsworth, (1943) *Journal of the Optical Society of America, 33*(10), 568–578

^(o)^Della Sala et al., (1996) *Archivio di Psicologia, Neurologia e Psichiatria, 57*, 327–342

^(p)^Spinnler, H. & Tognoni, G. (1987) *The Italian Journal of Neurological Sciences, 8*[Suppl], 1–120

^(q)^Smirni et al., *Neurological Sciences, 39*(8), 1391–1399

Full references of the neuropsychological tests are available in the electronic supplementary materials

### Famous people recognition assessment

The patient’s ability to recognize familiar people through personal face, name, and voice was tested on a range of tasks summarized in Table 2.

**Table 2:** Test of face and people recognition

	Cut-off	Raw score	Adjusted score
Facial Recognition Test (BFRT)^(a)^	< 37	43/54	46
Famous people recognition through face (FA-REC)^(b)^			
Face recognition: familiarity score	< 47.23	45/60	45.7*
Face recognition: semantic score	< 69.41	75/120	79.23
Face recognition: false alarm score	> 8.41	3/20†	2.4
Famous people recognition through voice (VO-REC)^(b)^			
Voice recognition: familiarity score	< 35.56	49/60	49.44
Voice recognition: semantic score	< 34.46	43/120	45.54
Voice recognition: false alarm score	> 8.5	0/20‡	0
Famous people recognition through personal name (NA-REC)^(c)^			
Name recognition: familiarity score	< 53.88	60/60	60
Name recognition: semantic score	< 86.67	120/120	80
Name recognition: false alarm score	> 1.97	0/20§	0
Recognition Memory Test—Faces^(d)^	< 21.59	17	17.12*
Ekman 60-Faces Test^(e)^	< 37.47	53	55.97
Total score	< 37.47	53/60	55.97
Surprise	< 6	9/10	
Happiness	< 9	10/10	
Fear	< 2	5/10	
Disgust	< 4	9/10	
Anger	< 5	10/10	
Sadness	< 4	10/10	

*Pathological scores

Raw scores are adjusted for age, for education, and, when indicated, for sex, according to the parameters estimated in a normal large sample with a multiple regression model. Adjusted scores < 5% one-sided non-parametric tolerance limit (with 95% CI) are considered pathological: inferential cut-off scores are therefore those at which or below which the probability that an individual belongs to the normal population is < 0.05

Bayesian Test for a Deficit allowing for Covariates (BTD-Cov), patient vs control group (*n* = 17):

†*p* = 0.127; Z-CCC = 1.719; Bayesian point estimate = 93.641%

‡*p* = 0.662; Z-CCC = −0.475; Bayesian point estimate = 33.087%

§*p* = 0.646; Z-CCC = −0.499; Bayesian point estimate = 32.316%

References for the neuropsychological tests

^(a)^Ferracuti et al., (2000) Florence, Italy, Giunti Organizzazioni Speciali

^(b)^Quaranta et al., (2016) *Neurological Sciences, 37*(3), 345–352

^(c)^Piccininni et al., (2018) *Neurological Sciences, 39*(4), 663–669

^(d)^Smirni et al., (2018) *Neurological Sciences, 39*(8), 1391–1399

^(e)^Dodich et al., (2014) *Neurological Sciences, 35*(7), 1015–1021

Full references of the neuropsychological tests are available in the electronic supplementary materials

#### People recognition from faces

The patient had no difficulties in an unfamiliar face matching test, ruling out the hypothesis of apperceptive prosopagnosia, although this test has been challenged [30]. In contrast, he performed very poorly in a famous face recognition test. This consists in 60 black-and-white photographs (40 famous faces, well-known at the national level, and 20 non-famous faces); the patient is first asked to provide a familiarity judgment (“is this face familiar to you?”). A false alarm score (range 0–20), namely, the number of unknown faces judged as familiar is also recorded. If the answer to the familiarity judgment is positive and correct, the participant is asked three further questions. The first two are multiple choice ones, exploring the general and specific categories to which famous persons belong. A general information would be: “is this person involved in (a) politics; (b) entertainment; (c) sport; (d) civil society?”. If the patient answers correctly, for example, (b) entertainment, a specific information is: “is this person involved in (a) cinema; (b) theatre; (c) music; (d) TV?”. The third question is open and requires the subject to provide univocally identifying information (i.e., movie titles, political roles/parties, etc.). One point is assigned to each correct answer (range 0–120). Finally, the patient is asked to name the person.

The familiarity score (obtained by summing the number of faces correctly identified as famous or non-famous) was very low. The patient did not experience any familiarity feeling in front of very well-known celebrities’ faces (e.g., anchorman Bruno Vespa) for whom none of the healthy subjects in a previous study [31] failed to feel familiarity (see Table 3).

**Table 3:** Percentage of the occurrence of the familiarity feeling in healthy subjects for each famous person for which the patient failed to feel familiarity feeling on the famous people recognition through face (FA-REC)

**Celebrities**	*N* (155)	%
Bruno Vespa	155	100
Luciana Littizzetto	154	99.4
Mara Venier	153	98.7
Francesco Totti	151	97.4
Pier Ferdinando Casini	151	97.4
Fabrizio Frizzi	150	96.8
Lilli Gruber	148	95.5
Pope Benedict XVI	147	94.8
Michele Santoro	143	92.3
Piero Angela	142	91.6
Patty Pravo	139	89.7
Andrea Bocelli	101	65.2

The table reports the number of healthy subjects (and the corresponding percentage of the sample) who reported a familiarity feeling for the celebrities from whom the patient failed to feel familiarity. The data in this table are adapted with permission from Piccininni et al. [31]

The patient provided semantic information for the celebrities he was able to recognize, suggesting that semantic knowledge was preserved.

#### People recognition from voice

The patient was asked to carefully listen to 60 audio fragments (15 s of neutral discourses) of the same 40 celebrities of the previous test and 20 non-famous voices. The procedure and scoring were the same as in the previous task.

The patient’s performance in voice recognition, a difficult task even for controls, was normal.

#### People recognition from name

The patient was asked to identify the same 40 celebrities (among 20 distractors) from written name. The procedure was the same as in the two previous tests. Even in this case, the patient’s performance was normal.

The order of presentation of faces, voices, and names was random and differed in the three versions of the test.

However, the selective face familiarity defect could be due to a bias that made the patient reluctant to report familiarity unless he was very confident.

In order to check this hypothesis, we ran the Bayesian Test for a Deficit allowing for Covariates (BTD-Cov [32]) comparing the false alarm scores on the three famous people recognition tests obtained by our patient against those obtained by 17 controls matched for age (*M* = 54.35 years; *SD* 2.308 years) and education (*M* = 10.26 years; *SD* 3.040 years). Since in the normative study education significantly affected the number of false alarms in the three tests, we covaried for education. There were no significant differences, suggesting that the patient’s behavior was the same for all tasks and comparable to that of controls (see Table 2 for the analyses).

### New face recognition

Prosopagnosic patients are poor in face learning [33] unless they are given shallow encoding instructions; therefore, we submitted this patient to a new face recognition task.

The test involves a study and a recognition stage. In the former, 30 target stimuli (black-and-white photographs of unfamiliar faces with neutral expression and no specific features) were individually displayed with a 3-s interval per item. In order to guarantee an adequate attentional level, the subject was instructed to judge the pleasantness of each face.

In the recognition phase, the patient had to recognize each target shown among two distractors (unfamiliar faces with similar physiognomic features).

The patient performed very poorly, showing that his difficulties involved also new face learning.

### Emotion recognition

Some prosopagnosic patients can recognize facial emotions (see [34] for review), but the absence of convincing dissociations has played a role in theories of face processing, e.g. [35].

We assessed this ability by means of the Italian version of the Ekman 60-Faces Test.

The patient’s performance was unremarkable for each of the six basic emotions and in the overall score (see Table 2 for tests concerning face and people recognition), demonstrating preserved facial emotion recognition.

### Recognition of famous buildings

To verify whether the deficit was limited to famous people or included other unique items, this patient was submitted to a famous building recognition task, which included 20 Italian and non-Italian items.

In contrast with his face recognition difficulties, the patient performed well, recognizing and naming 17 out of 20 famous buildings. Five matched controls produced a mean of 15 correct responses.

### New building recognition

In order to evaluate the specificity of the new face learning difficulties, we submitted the patient to an unfamiliar building recognition task. The procedure was the same as in the previous test, but stimuli were 30 black-and-white photos of buildings (with typically Italian architectural features, stylistic neutrality, absence of specific connotations, and verbal cues). The patient’s performance was normal, supporting the disorder specificity.

### Follow-up

The patient underwent a second examination 16 months later. The results of the follow-up are reported on Table 4.

**Table 4:** Follow-up neuropsychological assessment

	Cut-off	Raw score	Adjusted score
Test of face and people recognition			
Visual Recognition of Celebrities^(a)^	< 6325	4941.5/8001	4980.96*
Visuo-perceptual, visuo-spatial, and visuo-constructive abilities			
Birmingham Object Recognition Battery—BORB^(b)^			
Length match task	≤ 24	28/30	
Size match task	≤ 23	28/30	
Orientation match task	≤ 20	29/30	
Position of gap match task	≤ 27	36/40	
Naming of overlapping letters: paired overlapping/non-overlapping	> 1.2	1.02	
Naming of overlapping letters: triplets overlapping/non-overlapping	> 1.0	1.0	
Naming of overlapping shapes: paired overlapping/non-overlapping	> 1.0	0.99	
Naming of overlapping shapes: triplets overlapping/non-overlapping	> 1.0	1.0	
Naming of overlapping drawings: paired overlapping/non-overlapping	> 1.3	0.98	
Minimal feature view task	≤ 19	25/25	
Foreshortened view task	≤ 16	25/25	
Object decision task—OD B easy	≤ 28	32/32	
Object decision task—OD A hard	≤ 23	31/32	
Item match task	≤ 26	32/32	
Associative match task	≤ 22	30/30	
Picture naming	≤ 8	14/15	
Picture naming (mean response time per item in sec.)		1.24	
Picture naming (total response time in sec.)		24.40	

*Pathological scores

Raw scores are adjusted for age, for education, and, when indicated, for sex, according to the parameters estimated in a normal large sample with a multiple regression model. Adjusted scores < 5% one-sided non-parametric tolerance limit (with 95% CI) are considered pathological: inferential cut-off scores are therefore those at which or below which the probability that an individual belongs to the normal population is < 0.05

References for the neuropsychological tests

^(a)^Bizzozero et al., (2005) *Neurological Sciences, 26*(2), 95–107

^(b)^Humphreys & Riddoch, J. M. (1993) Hove, UK, Lawrence Erlbaum

Full references of the neuropsychological tests are available in the electronic supplementary materials

Although he had resumed his previous social life, he still complained about difficulties in recognizing people. Since he remembered the people he did not recognize in the previous examination, we used a different version of famous face recognition. This task includes 126 13 × 20 cm, black-and-white photographs—63 belonging to celebrities and 63 to unknown people—and requires a familiarity judgment, followed by identification (providing semantic information about the correctly recognized people): participants answer two multiple-choice questions concerning the celebrity’s period of fame and his/her professional category and one open question asking for any further information. Identification is assessed sequentially and only for faces correctly judged as famous. Then, the participant is required to name the item.

As faces are not equally difficult to recognize, the scoring procedure is based on a rank order score. The difficulty of each item was determined according to the number of the participants’ failures with each individual face. The faces were then ranked from the most difficult, i.e., those which yielded the largest number of failures, to the easiest ones (smallest number of errors). A rank score of 1.0 was assigned to the most difficult items and of 12.0 to the easiest ones. The patient’s performance was well below the cut-off. In particular, he did not identify very popular Italian people, such as Rita Levi Montalcini (identified by 91/98 controls) or Piero Angela (142/155 controls).

We also re-tested the patient on unknown faces, recording response times, in order to verify whether his correct performance required an increased amount of time.

The original items of the long form of the Benton Facial Recognition Test were scanned and presented in a computerized format (MATLAB version R2019b). The panel/items were presented in a randomized order, and the patient was asked to respond as accurately and fast as possible by pressing the corresponding number on the keyboard. Both the target and the probe faces subtended an angle of 7° × 7° when viewed from 60 cm. Each panel remained on the computer screen until the patient completed the response or for a maximum of 30 s, without any constraint regarding the order of response for items requiring three choices and without the possibility to deselect a face. After each panel a black screen was presented for 3 s. The procedure of this computerized version was similar to a previous one [28], but, due to some differences between the two versions and the age of the samples, we collected new control data.

The patient’s accuracy was 44 out of 54, well above the cut-off. The mean response time for item was 6.60 s. Six control participants matched for age (*M* = 56; *SD* 3.688) and educational level (*M* = 10; *SD* 1.55) obtained a mean accuracy score of 43.17 (*SD* 3.06, range 41–48), while their mean response time for item was 6.50 s (*SD* 1.19). Both, accuracy and response time were not significantly different (see Table 5).

**Table 5:** Results on the two computerized tasks

	Control group score (M ± SD)	Patient’s raw score	Patient’s Z-score
Computerized Benton Facial Recognition Test (BFRT)			
Accuracy (0–54)	43.17 ± 3.06	44†	0.27
Total reaction time (sec.)	350.99 ± 64.42	356.45	0.08
Mean response time per item (sec.)	6.50 ± 1.19	6.60‡	0.08
Computerized picture-naming test			
Accuracy (0–15)	14.17 ± 0.41	14 §	−0.41
Total reaction time (sec.)	24.40 ± 3.85	18.59	−1.51
Mean response time per item (sec.)	1.63 ± 0.26	1.24 |	−1.50

Bayesian Test for a Deficit allowing for Covariates (BTD-Cov), patient vs control group (*n* = 6):

†*p* = 0.863; Z-CCC = 0.272; Bayesian point estimate = 56.825%

‡*p* = 0.957; Z-CCC = 0.085; Bayesian point estimate = 52.130%

§*p* = 0.796; Z-CCC = − 0.409; Bayesian point estimate = 39.804%

|*p* = 0.375; Z-CCC = − 1.508; Bayesian point estimate = 18.769%

Finally, the patient performed the BORB perfectly, even with triplets of overlapping items. In particular, the original items of the short version of the picture-naming task (low frequency animate and inanimate drawings) were scanned and presented in a computerized format using MATLAB version R2019b. The drawings were presented in the same fixed order as in the original version, and the patient was asked to name the drawings as accurate and fast as possible.

There were no significant differences in accuracy and response time (see Table 5).

## Discussion

We described a patient with a persistent deficit in face recognition, representing a mild form of prosopagnosia due to a left temporo-occipital lesion. This case presents unexpected features. First, a deficit in face familiarity is observed after bilateral or RH lesions [17, 18, 36]; moreover, patients with left temporo-occipital lesions usually show associative visual agnosia or a more general semantic disorder [17, 37, 38], while our patient was not agnosic for objects and had normal semantics for famous people. Topographical disorientation and dyschromatopsia were absent.

Secondly, while familiarity feelings are relatively or completely spared in left-brain-damaged patients [14, 16, 17], our patient denied any familiarity feeling even with very well-known celebrities, similar to right-brain-damaged prosopagnosic people.

The interpretation of these findings is not univocal. According to Barton [16], cases of prosopagnosia after left-sided lesions in left-handed subjects could be attributed to a reversed hemispheric specialization for face processing. A partly similar explanation of data in the literature and of our patient could be based on De Renzi et al.’s [3] assumption that hemispheric specialization for face processing may be a graded phenomenon. De Renzi et al. [3] assumed that right-handers differ in their degree of RH specialization in processing faces, and in only a minority of them, this asymmetry is so marked that it cannot be compensated for by the healthy LH. If this model is correct and face recognition is asymmetrically subserved by both hemispheres, then prosopagnosic patients should be distributed according to a Gaussian curve, where the highest number of subjects has bilateral lesions, a large minority RH damage, and a small minority LH lesions. The distribution of prosopagnosic patients according to lesion laterality [39] is consistent with this prediction. A second prediction based on this model is that handedness should allow identifying prosopagnosic patients with LH lesions. Consistent with this is the observation that 3 out of 4 prosopagnosic left-brain-damaged patients reported in the literature were left-handers. Also consistent with these expectations are the high proportion of left-handedness in prosopagnosic patients with a less clear evidence of lesions restricted to the LH [17] and our patient’s left-handedness familiarity. Szaflarski et al. [40] showed that both personal handedness and a family history are equally associated with the language laterality index. A last expectation could be that the severity of face recognition disorders should be rather mild in prosopagnosic patients with LH lesions. If hemispheric specialization for face processing is a graded phenomenon, in patients with a lesion restricted to the LH, prosopagnosia should be not only less frequent but also less severe. Even though data gathered in prosopagnosic patients with intact RH are too heterogeneous to check it, the relatively mild disorder of our patient could be rather consistent with this prediction. However, Subject 015 of Barton [16], with a moderately severe defect in face familiarity, scored better than chance on a forced-choice version of the test, suggesting a spared covert familiarity feeling.

The claim that our patient’s face recognition disorders were due to a selective defect of face familiarity is documented also by his performance on the new face recognition task. These selective defects of face familiarity are difficult to explain because not only face familiarity feelings seem spared in left-brain-damaged patients [17], but they have also been linked to the right temporal lobe in a recent study on the neuroanatomical substrates of overt face processing [18].

Another interesting point was that, despite a lesion involving the left OFA, the patient performed well with unknown face recognition. This could be attributed to an intact right OFA that allows processing of perceptual features. Re-entrant connections and dynamic interactions between different structures involved in face recognition have, indeed, been proposed by different authors [41–43]. An interaction between normal processing of perceptual features by the intact right OFA and acknowledgement by the left FFA that the corresponding face is actually unknown could, therefore, allow to explain this unexpected finding.

The main limitation of our study is that the patient had a mild form of prosopagnosia that suggests caution in interpreting results. Finally, we could not discuss his implicit recognition, since we recorded skin conduction during face presentation, but, due to technical reasons, these data were unreliable.

## Electronic supplementary material

ESM 1 (DOCX 19 kb).
