# Supplementary material for: Selective defects of face familiarity associated to a left temporo-occipital lesion
Source: Neurol Sci. 2020 Jul 10;42(2):613–23. doi: 10.1007/s10072-020-04581-5 (PMC7843582; doi:10.1007/s10072-020-04581-5)
Supplement: Supplementary file 1 — (DOCX 19 kb). [file 10072_2020_4581_MOESM1_ESM.docx]

**Selective defects of face familiarity associated to a left temporo-occipital lesion.**

**Abstract**

Acquired prosopagnosia is usually a consequence of bilateral or right hemisphere lesions and is often associated with topographical disorientation and dyschromatopsia. Left temporo-occipital lesions sometimes result in a face recognition disorder but in a context of visual object agnosia with spared familiarity feelings for faces, usually in left-handers. We describe a patient with a left temporo-occipital hemorrhagic lesion unexpectedly resulting in a deficit of face familiarity, which could represent a mild form of associative prosopagnosia. Our patient failed to feel familiarity feelings even with very well-known famous faces but had neither visual object agnosia nor defects with semantics or naming of celebrities. This was confirmed even when the patient was re-tested a year later. We speculate that a graded lateralization of face processing could be at the basis of occasional cases of prosopagnosia.

**Introduction**

Acquired prosopagnosia is a disorder of visual recognition specific to faces, associated with occipital or temporal bilateral lesions; occasionally, damage is restricted to the right hemisphere (RH)[1; 2], as reported in a review with 27 cases with neuroimaging plus four cases with surgical evidence of association between prosopagnosia and RH damage only [3]. An RH superiority in face processing [4] is confirmed by visual hemifield experiments [5-7], activation studies [e.g., 8, 9], EEG scalp topography [e.g., 10], TMS over the right **occipital face area** (OFA) [e.g., 11], and intracranial stimulation [e.g., 12].

Despite this converging evidence, support to a left hemisphere (LH) contribution comes from neuroimaging and clinical findings. All the cited functional neuroimaging studies, though revealing that face perception results in a greater activation in right-sided face-processing network, show face-selective activation in the left fusiform region. Concerning clinical findings, there are four cases of prosopagnosia with LH lesions and intact RH [13-16]. Three of them [13, 14 and 16] were left-handed. More patients (with less evidence of restricted LH lesions) have been described (see [17] for review). Some qualitative features allow distinguishing the rare instances of left temporo-occipital lesions causing face recognition defects from the more frequent cases of right homologous lesions. These features concerned: (a) high proportion of left-handedness; (b) relative or complete sparing of familiarity feelings; (c) coexistence of visual object agnosia. The explanation has been a defective ability to access both conceptual and person-specific semantic information from visual modality. The lack of familiarity found in right brain-damaged patients contrasts with its preservation after left brain-damage [17]. The relations between loss of face familiarity feelings and disruption of RH structures have been confirmed [18], studying, in a large sample of neurodegenerative patients, the neuroanatomical substrates of three steps of famous face processing, namely: (a) familiarity judgment; (b) semantic/biographical information retrieval and (c) naming. Familiarity correlated with right anterior middle temporal gyrus integrity, whereas performance in naming and semantic information retrieval significantly correlated with grey matter volume in the left anterior temporal lobe.

Most prosopagnosia cases are due to lesions of a bilateral network spanning from the inferior occipital gyrus, corresponding to the OFA [19], to the mid-fusiform gyrus, where the face fusiform area is (FFA; [20]), to the anterior temporal cortex (the AT of [21], or aIT of [22]). The inferior occipital areas mainly subsume the first stages of face perception [23], whereas a recognition-driven activity is carried out in FFA and aIT. Disconnection can cause a slightly different prosopoagnosic picture [24], with intact perceptual face encoding and face memories. Probes of perceptual encoding generally involve match-to-sample or discrimination tasks.

Prosopagnosia can be associated with hemianopia, topographical skills impairment [25], word recognition deficits [26], achromatopsia [27], and visual agnosia (see [28]).

We studied a case of face familiarity loss with neuroimaging evidence of a left temporo-occipital lesion without the features typical of patients with face recognition defects from left temporo-occipital lesions [17]. Paradoxically, in this patient only face familiarity feelings for famous people were selectively impaired, whereas semantic information retrieval and naming of people judged as familiar were intact. The selective impairment of mechanisms involved in familiarity was confirmed by the pathological score on face learning. A further interesting aspect was that, even if the lesion affected the OFA, which is regarded as involved in the fine-grained individual face analysis, he correctly matched unfamiliar faces. We thought, therefore, that a detailed report of this patient could be interesting, due to the variety of issues raised.

**Case report**

A 56-year old right-handed (with a left-handed brother) retired driver with 11 years of schooling came to our observation in May 2018 because of a right hemifield visual defect and calculation problems.

In February 2018, due to a left carotid aneurysm, the patient underwent an embolization procedure and was discharged with a triple anti-platelet therapy. He reported mild word finding difficulties for three days, that spontaneously recovered. Ten days later, he suddenly claimed written language difficulties with spontaneous recovery. On March 4th, the patient woke up with right homonymous hemianopia and was admitted to the emergency department of the local hospital, where the neurological examination revealed only hemianopia. A CT scan showed two intracerebral hemorrhages, a recent one in the left occipital lobe and a sub-acute (compatible with the reported written language and calculation difficulties) in the left parietal region. An MRI confirmed the two lesions (see Figure 1).

---Insert Figure 1 about here---

The patient also complained impairment in recognizing people unless they spoke. Therefore, we investigated this ability in detail, after obtaining his written informed consent.

General cognitive assessment

**For all the tests used with this patient, normative data are available: raw scores are adjusted for age, education and, when indicated, for sex, according to the parameters estimated in a normal sample (200–321 neurologically unimpaired subjects) with a multiple regression model (see [29] for an extensive explanation of the standardization procedure). Adjusted scores 55% one-sided non-parametric tolerance limit (with 95% CI) are considered pathological: inferential cut-off scores are therefore those at which or below which the probability that an individual belongs to the normal population is <0.05.**

On an extensive neuropsychological battery (see Table 1 **and Supplementary Material**) performed in our Cognitive Neurorehabilitation Center by a neuropsychologist, the patient showed no deficits except for mild difficulties with calculation. In particular, his performance was errorless in naming 48 objects pertaining to different living and non-living categories, ruling out also visual agnosia.

---Insert Table 1 about here---

Famous people recognition assessment

The patient’s ability to recognize familiar people through personal face, name, and voice was tested on a range of tasks summarized in Table 2.

---Insert Table 2 about here---

*People recognition from faces*. The patient had no difficulties in an unfamiliar face matching test, ruling out the hypothesis of apperceptive prosopagnosia, although this test has been challenged [30]. In contrast, he performed very poorly in a famous face recognition test. This consists in 60 black-and-white photographs (40 famous faces, well-known at the national level, and 20 non-famous faces); the patient is first asked to provide a familiarity judgment (‘‘is this face familiar to you?’’). A false alarm score (range 0–20), namely the number of unknown faces judged as familiar is also recorded. If the answer to the familiarity judgment is positive and correct, the participant is asked three further questions. The first two are multiple choice ones, exploring the general and specific categories to which famous persons belong. A general information would be: ‘‘is this person involved in (a) politics; (b) entertainment; (c) sport; (d) civil society?’’. If the patient answers correctly, for example (b) entertainment, a specific information is: ‘‘is this person involved in (a) cinema; (b) theatre; (c) music; (d) TV?’’. The third question is open and requires the subject to provide univocally identifying information (i.e., movie titles, political roles/parties, etc.). One point is assigned to each correct answer (range 0–120). Finally, the patient is asked to name the person.

The familiarity score (obtained by summing the number of faces correctly identified as famous or non-famous) was very low. The patient did not experience any familiarity feeling in front of very well-known celebrities’ faces (e.g. anchor-man Bruno Vespa) for whom none of the healthy subjects in a previous study [31] failed to feel familiarity (see Table 3).

--- Insert Table 3 around here---

The patient provided semantic information for the celebrities he was able to recognize, suggesting that semantic knowledge was preserved.

*People recognition from voice*. The patient was asked to carefully listen to 60 audio fragments (15 s of neutral discourses) of the same 40 celebrities of the previous test and 20 non-famous voices. The procedure and scoring were the same as in the previous task.

The patient’s performance in voice recognition, a difficult task even for controls, was normal.

*People recognition from name*

The patient was asked to identify the same 40 celebrities (among 20 distractors) from written name. The procedure was the same as in the two previous tests. Even in this case, the patient’s performance was normal.

The order of presentation of faces, voices and names was random, and differed in the three versions of the test.

However, the selective face familiarity defect could be due to a bias that made the patient reluctant to report familiarity unless he was very confident.

In order to check this hypothesis, we ran the Bayesian Test for a Deficit allowing for Covariates (BTD-Cov [32]) comparing the false alarm scores on the three famous people recognition tests obtained by our patient against those obtained by 17 controls matched for age (*M*=54.35 yrs; *SD* 2.308 yrs) and education (*M*=10.26 yrs; *SD* 3.040 yrs). Since in the normative study education significantly affected the number of false alarms in the three tests, we covaried for educational. There were no significant differences, suggesting that the patient’s behavior was the same for all tasks and comparable to that of controls (see Table 2 for the analyses).

New face recognition

Prosopagnosic patients are poor in face learning [33] unless they are given shallow encoding instructions; therefore, we submitted this patient to a new face recognition task.

The test involves a study and a recognition stage. In the former, 30 target stimuli (black and white photographs of unfamiliar faces with neutral expression and no specific features) were individually displayed with a 3 s interval per item. In order to guarantee an adequate attentional level, the subject was instructed to judge the pleasantness of each face.

In the recognition phase, the patient had to recognize each target shown among two distractors (unfamiliar faces with similar physiognomic features).

The patient performed very poorly, showing that his difficulties involved also new face learning.

Emotion recognition

Some prosopagnosic patients can recognize facial emotions (see [34] for review), but the absence of convincing dissociations has played a role in theories of face processing [e.g., 35].

We assessed this ability by means of the Italian version of the Ekman 60-Faces Test.

The patient's performance was unremarkable for each of the six basic emotions and in the overall score (see Table 2 for tests concerning face and people recognition), demonstrating preserved facial emotion recognition.

Recognition of famous buildings

To verify whether the deficit was limited to famous people or included other unique items, this patient was submitted to a famous building recognition task, which included 20 Italian and non-Italian items.

In contrast with his face recognition difficulties, the patient performed well, recognizing and naming 17 out of 20 famous buildings. Five matched controls produced a mean of 15 correct responses.

New building recognition

In order to evaluate the specificity of the new face learning difficulties, we submitted the patient to an unfamiliar building recognition task. The procedure was the same as in the previous test, but stimuli were 30 black and white photos of buildings (with typically Italian architectural features, stylistic neutrality, absence of specific connotations and verbal cues). The patient’s performance was normal, supporting the disorder specificity.

Follow-up

The patient underwent a second examination 16 months later. The results of the follow-up are reported on Table 4.

Although he had resumed his previous social life, he still complained about difficulties in recognizing people. Since he remembered the people he did not recognize in the previous examination, we used a different version of famous face recognition. This task includes 126,13x20 cm, black-and-white photographs – 63 belonging to celebrities and 63 to unknown people, and requires a familiarity judgement, followed by identification (providing semantic information about the correctly recognized people): participants answer two multiple-choice questions concerning the celebrity’s period of fame and his/her professional category, and one open question asking for any further information. Identification is assessed sequentially and only for faces correctly judged as famous. Then, the participant is required to name the item.

As faces are not equally difficult to recognize, the scoring procedure is based on a rank order score. The difficulty of each item was determined according to the number of the participants’ failures with each individual face. The faces were then ranked from the most difficult, i.e., those which yielded the largest number of failures, to the easiest ones (smallest number of errors). A rank score of 1.0 was assigned to the most difficult items and of 12.0 to the easiest ones. The patient’s performance was well below the cut-off. In particular, he did not identify very popular Italian people, such as Rita Levi Montalcini (identified by 91/98 controls) or Piero Angela (142/155 controls).

We also re-tested the patient on unknown faces, recording response times, in order to verify whether his correct performance required an increased amount of time.

The original items of the long form of the Benton Facial Recognition Test were scanned and presented in a computerized format (Matlab version R2019b). The panel/items were presented in a randomized order and the patient was asked to respond as accurately and fast as possible by pressing the corresponding number on the keyboard. Both the target and the probe faces subtended an angle of 7° x 7° when viewed from 60cm. Each panel remained on the computer screen until the patient completed the response or for a maximum of 30 seconds, without any constraint regarding the order of response for items requiring three choices and without the possibility to deselect a face. After each panel a black screen was presented for 3 seconds. The procedure of this computerized version was similar to a previous one [28] but, due to some differences between the two versions and the age of the samples, we collected new control data.

The patient’s accuracy was 44 out of 54, well above the cut-off. The mean response time for item was 6.60 s. Six control participants matched for age (*M*=56; *SD* 3.688) and educational level (*M*=10; *SD* 1,55) obtained a mean accuracy score of 43.17 (*SD* 3.06, range 41-48), while their mean response time for item was 6.50 s (*SD* 1.19). Both, accuracy and response time, were not significantly different (see Table 5).

Finally, the patient performed the BORB perfectly, even with triplets of overlapping items. In particular, the original items of the short version of the picture naming task (low frequency animate and inanimate drawings) were scanned and presented in a computerized format using Matlab version R2019b. The drawings were presented in the same fixed order as in the original version and the patient was asked to name the drawings as accurate and fast as possible.

There were no significant differences in accuracy and response time (see Table 5).

---- Insert Tables 4 and 5 about here-----

**Discussion**

We described a patient with a persistent deficit in face recognition, representing a mild form of prosopagnosia due to a left temporo-occipital lesion. This case presents unexpected features. First, a deficit in face familiarity is observed after bilateral or RH lesions [17-18, 36]; moreover, patients with left temporo-occipital lesions usually show associative visual agnosia or a more general semantic disorder [17, 37-38] while our patient was not agnosic for objects and had normal semantics for famous people. Topographical disorientation and dyschromatopsia were absent.

Secondly, while familiarity feelings are relatively or completely spared in left-brain damaged patients [14, 16-17], our patient denied any familiarity feeling even with very well-known celebrities, similar to right-brain damaged prosopagnosic people.

The interpretation of these findings is not univocal. According to Barton [16] cases of prosopagnosia after left-sided lesions in left-handed subjects could be attributed to a reversed hemispheric specialization for face processing. A partly similar explanation of data in the literature and of our patient could be based on De Renzi et al.’s [39] assumption that hemispheric specialization for face processing may be a graded phenomenon. De Renzi et al. [39] assumed that right-handers differ in their degree of RH specialization in processing faces, and in only a minority of them this asymmetry is so marked that cannot be compensated for by the healthy LH. If this model is correct and face recognition is asymmetrically subserved by both hemispheres, then prosopagnosic patients should be distributed according to a Gaussian curve, where the highest number of subjects has bilateral lesions, a large minority RH damage and a small minority LH lesions. The distribution of prosopagnosic patients according to lesion laterality [40] is consistent with this prediction. A second prediction based on this model is that handedness should allow identifying prosopagnosic patients with LH lesions. Consistent with this is the observation that 3 out of 4 prosopagnosic left brain-damaged reported in the literature were left-handers. Also consistent with these expectations are: - the high proportion of left-handedness in prosopagnosic patients with a less clear evidence of lesions restricted to the LH [17] and - our patient’s left-handedness familiarity. Szaflarski et al. [41] showed that both personal handedness and a family history are equally associated with the language laterality index. A last expectation could be that the severity of face recognition disorders should be rather mild in prosopagnosic patients with LH lesions. If hemispheric specialization for face processing is a graded phenomenon, in patients with a lesion restricted to the LH, prosopagnosia should be not only less frequent, but also less severe. Even though data gathered in prosopagnosic patients with intact RH are too heterogeneous to check it, the relatively mild disorder of our patient could be rather consistent with this prediction. However, Subject 015 of Barton [16] with a moderately severe defect in face familiarity, scored better than chance on a forced-choice version of the test, suggesting a spared covert familiarity feeling.

The claim that our patient’s face recognition disorders were due to a selective defect of face familiarity is documented also by his performance on the new face recognition task. These selective defects of face familiarity are difficult to explain because not only face familiarity feelings seem spared in left-brain damaged patients [17], but they have also been linked to the right temporal lobe in a recent study on the neuroanatomical substrates of overt face processing [18].

Another interesting point was that, despite a lesion involving the left OFA, the patient performed well with unknown face recognition. This could be attributed to an intact right OFA that allows processing of perceptual features. **Re-entrant connections and dynamic interactions between different structures involved in face recognition have, indeed, been proposed by different authors [42-44]. An interaction between normal processing of perceptual features by the intact right OFA and acknowledgement by the left FFA that the corresponding face is actually unknown could, therefore, allow to explain this unexpected finding**.

**The main limitation of our study is that the patient had a mild form of prosopoagnosia that suggests caution in interpreting results. Finally, we could not discuss his implicit recognition, since we recorded skin conduction during face presentation but, due to technical reasons, these data were unreliable.**

**References**

1. De Renzi E. (1997). Prosopagnosia. In: T.E. Feinberg, M.J. Farah (Eds.), Behavioral neurology and neuropsychology, McGraw-Hill, New York, pp. 245-256.
2. Barton, J. J. (2008). Structure and function in acquired prosopagnosia: lessons from a series of 10 patients with brain damage. *Journal of Neuropsychology, 2*(Pt 1), 197–225.
3. De Renzi, E., Perani, D., Carlesimo, G. A., Silveri, M. C., & Fazio, F. (1994). Prosopagnosia can be associated with damage confined to the right hemisphere—An MRI and PET study and a review of the literature. *Neuropsychologia, 32*(8), 893–902.
4. Hécaen, H., & Angelergues, R. (1962). Agnosia for faces (Prosopoagnosia*). Archives of Neurology, 7*, 92-100.
5. Sergent, J., Bindra D. (1981). Differential hemispheric processing of faces: Methodological considerations and reinterpretation. *Psychological Bulletin, 89*(3) :541-554.
6. Rhodes, D. (1985). Lateralized processes in face recognition. *British Journal of Psychology, 76*(2) (1985), pp. 249-271
7. Levine, S. C., Banich, M. T., Koch-Weser, M.P. (1988). Face recognition: A general or specific right hemisphere capacity? *Brain and Cognition, 8*(3), 303-325.
8. Rossion, B. (2008). Constraining the cortical face network by neuroimaging studies of acquired prosopagnosia. *NeuroImage, 40*(2), 423–426. https://doi.org/10.1016/j.neuroimage.2007.10.047.
9. Yovel, G., Kanwisher, N. (2004). Face perception: domain specific, not process specific. *Neuron, 44*, 747–748.
10. Rossion, B, & Boremanse, A. (2011). Robust sensitivity to facial identity in the right human occipito-temporal cortex as revealed by steady-state visual evoked potentials. *Journal of Vision, 11*(2), 16.
11. Pitcher, D., Walsh, V., Yovel, G., Duchaine, B. (2007). TMS evidence for the involvement of the right occipital face area in early face processing. *Current Biology, 17*, 1568–1573.
12. Jonas, J., Brissart, H., Hossu, G., Colnat-Coulbois, S., Vignal, J.-P., Rossion, B., & Maillard, L. (2018). A face identity hallucination (palinopsia) generated by intracerebral stimulation of the face-selective right lateral fusiform cortex. *Cortex, 99*, 296-310.
13. Eimer, M., & McCarthy, R.A. (1999). Prosopagnosia and structural encoding of faces: evidence from event related potentials*. Neuroreport, 10* (2), 255-259.
14. Mattson, A. J., Levin, H. S., & Grafman, J. (2000). A Case of Prosopagnosia Following Moderate Closed Head Injury with Left Hemisphere Focal Lesion. *Cortex, 36*(1), 125–137. <https://doi.org/10.1016/S0010-9452(08)70841-4>.
15. Wright, H., Wardlaw, J., Young, A. W., & Zeman, A. (2006). Prosopagnosia following nonconvulsive status epilepticus associated with a left fusiform gyrus malformation. *Epilepsy & Behavior: E&B, 9*(1), 197–203. <https://doi.org/10.1016/j.yebeh.2006.04.010>.
16. Barton, J.J. (2008). Prosopagnosia associated with a left occipitotemporal lesion. *Neuropsychologia, 46*(8):2214-2224. doi: 10.1016/j.neuropsychologia.2008.02.014.
17. Gainotti, G., & Marra, C. (2011). Differential Contribution of Right and Left Temporo-Occipital and Anterior Temporal Lesions to Face Recognition Disorders. *Frontiers in Human Neuroscience, 5*. <https://doi.org/10.3389/fnhum.2011.00055>
18. Borghesani, V, Narvid, J., Battistella, G., Shwe, W., Watson, C., Binney, R.J., Sturm, V., Miller, Z., Mandelli, M.L., Miller, B., Gorno-Tempini, M.L. (2019) Looks familiar, but I do not know who she is”: The role of the. anterior right temporal lobe in famous face recognition. *Cortex, 115*, 72-85. <https://doi.org/10.1016/j.cortex.2019.01.006>
19. Gauthier I, Tarr MJ, Moylan J Skudlarski P, Gore JC, Anderson AW. (2000). The fusiform “face area” is part of a network that processes faces at the individual level. *Journal of Cognitive Neuroscience, 12*, 495–504. DOI: 10.1162/089892900562165.
20. Kanwisher, N., McDermott, J., Chun, M.M. (1997). The fusiform face area: a module in human extrastriate cortex specialized for face perception. *The Journal of Neuroscience: the Official Journal of the Society for Neuroscience,17*(11), 4302-4311. DOI: 10.1523/JNEUROSCI.17-11-04302.1997.
21. Nasr, S., & Tootell, R.B.H. (2012). Role of fusiform and anterior temporal cortical areas in facial recognition. *Neuroimage, 63*, 1743-1753. DOI: 10.1016/j.neuroimage.2012.08.03.
22. Kriegeskorte N, Formisano E, Sorger B, Goebel R. (2007). Individual faces elicit distinct response patterns in human anterior temporal cortex. *Proc Natl Acad Sci USA, 104*(51), 20600-5. DOI: 10.1073/pnas.0705654104.
23. Mattavelli, G., Rosanova, M., Casali, A.G., Papagno, C., Romero Lauro, L.J. (2013) Top-down interference and cortical responsiveness in face processing: A TMS-EEG study. *NeuroImage, 76*, 24-32.
24. Fox, C., Iaria, G., & Barton, J. (2008). Disconnection in prosopagnosia and face processing. *Cortex, 44*(8), 996–1009. <https://doi.org/10.1016/j.cortex.2008.04.003>.
25. Corrow, J. C., Corrow, S. L., Lee, E., Pancaroglu, R., Burles, F., Duchaine, B., … Barton, J. J. S. (2016). Getting lost: Topographic skills in acquired and developmental prosopagnosia. *Cortex; a Journal Devoted to the Study of the Nervous System and Behavior, 76*, 89–103. <https://doi.org/10.1016/j.cortex.2016.01.003>
26. Behrmann, M., & Plaut, D. C. (2014). Bilateral Hemispheric Processing of Words and Faces: Evidence from Word Impairments in Prosopagnosia and Face Impairments in Pure Alexia. *Cerebral Cortex, 24*(4), 1102–1118. <https://doi.org/10.1093/cercor/bhs390>.
27. Moroz, D., Corrow, S. L., Corrow, J. C., Barton, A. R. S., Duchaine, B., & Barton, J. J. S. (2016). Localization and patterns of Cerebral dyschromatopsia: A study of subjects with prospagnosia. *Neuropsychologia, 89*, 153–160. <https://doi.org/10.1016/j.neuropsychologia.2016.06.012>.
28. Rossion, B. and Michel, C. (2018) Normative accuracy and response time data for the computerized Benton Facial Recognition Test (BFRT-c). *Behavior Research Methods, 50*(6), 2442-2460.
29. **Capitani, E. (1997). Normative data and neuropsychological assessment, common problems in clinical practice and research. *Neuropsychological Rehabilitation*, *7*(4), 295–309.** [**https://doi.org/10.1080/713755543**](https://doi.org/10.1080/713755543)
30. Duchaine, B., & Nakayama, K. (2006). The Cambridge Face Memory Test: results for neurologically intact individuals and an investigation of its validity using inverted face stimuli and prosopagnosic participants. *Neuropsychologia, 44*(4), 576–585. <https://doi.org/10.1016/j.neuropsychologia.2005.07.001>.
31. Piccininni, C., Gainotti, G., Trojano, L., Luzzi, S., Papagno, C., Carlesimo, G. A., … Quaranta, D. (2017). Item consistency in retrieving person-specific semantic information from faces and voices: An exploratory study in healthy subjects. *Visual Cognition, 25*(4–6), 679–689. <https://doi.org/10.1080/13506285.2017.1344340>.
32. Crawford, J. R., Garthwaite, P. H., & Ryan, K. (2011). Comparing a single case to a control sample: Testing for neuropsychological deficits and dissociations in the presence of covariates. *Cortex, 47*, 1166-1178. DOI: 10.1016/j.cortex.2011.02.017.
33. Polster, M. R., & Rapcsak, S. Z. (1996). Representations in learning new faces: evidence from prosopagnosia. *Journal of the International Neuropsychological Society: JINS, 2*(3), 240–248. DOI: 10.1017/s1355617700001181.
34. Nachson, I. (1995). On the modularity of face recognition: The riddle of domain specificity. *Journal of Clinical and Experimental* *Neuropsychology, 17*(2), 256-275. DOI: 10.1080/01688639508405122.
35. Calder, A.J., & Young, A.W. (2005). Understanding the recognition of facial identity and facial expression. *Nature reviews Neuroscience. 6*(8), 641-651.
36. **Carlesimo, G.A. & Caltagirone, C. (1995). Components in the visual processing of known and unknown faces. *Journal of Clinical and Experimental Neuropsychology* 17(5), 691-705**
37. **Damasio A., Tranel D., Damasio H. (1988). “Deep prosopagnosia”: A new form of acquired face recognition defect caused by left hemisphere damage. *Neurology* 38 (Suppl.1), 72**
38. **De Renzi E. (2000). Disorders of visual recognition. *Seminars in Neurology* 20(4), 479-85.**
39. De Renzi, E., Perani, D., Carlesimo, G. A., Silveri, M. C., & Fazio, F. (1994). Prosopagnosia can be associated with damage confined to the right hemisphere—An MRI and PET study and a review of the literature. *Neuropsychologia, 32*(8), 893–902. <https://doi.org/10.1016/0028-3932(94)90041-8>.
40. Corrow S, Dalrymple K, Barton J (2016). Prosopagnosia: current perspectives. *Eye and Brain,8*,165-175. doi: 10.2147/EB.S92838.
41. Szaflarski, JP, Binder JR, Possing ET, McKiernan KA, Ward BD, Hammeke TA. (2002) Language lateralization in left-handed and ambidextrous people: fMRI data. *Neurology, 59*(2), 238-244. DOI: <https://doi.org/10.1212/WNL.59.2.238>.
42. **Rossion B, Caldara R, Seghier M, Schuller AM, Lazeyras F, Mayer E. (2003). A network of occipito-temporal face-sensitive areas besides the right middle fusiform gyrus is necessary for normal face processing. Brain, 126(11), 2381-2395.**
43. **Righart R, Andersson F, Schwartz S, Mayer E, Vuilleumier P. (2010). Top-down activation of fusiform cortex without seeing faces in prosopagnosia. *Cerebral Cortex*, 20(8), 1878-1890**
44. **Barton, J., Corrow, R. (2016). Selectivity in Acquired Prosopagnosia: The Segregation of Divergent and Convergent Operations. *Neuropsychologia*, 83, 76-87.**
45. Engell, A. D., & McCarthy, G. (2013). Probabilistic atlases for face and biological motion perception: An analysis of their reliability and overlap. *Neuroimage, 74*, 140–151. <https://doi.org/10.1016/j.neuroimage.2013.02.025>.
46. Schmidt, P., Gaser, C., Arsic, M., Buck, D., Förschler, A., Berthele, A., … Mühlau, M. (2012). An automated tool for detection of FLAIR-hyperintense white-matter lesions in Multiple Sclerosis*. Neuroimage, 59*(4), 3774–3783. <https://doi.org/10.1016/j.neuroimage.2011.11.032>
47. Hua K, Zhang J, Wakana S, Jiang H, Li X, Reich DS, Calabresi PA, Pekar JJ, van Zijl PC, Mori S. (2008). Tract probability maps in stereotaxic spaces: Analyses of white matter anatomy and tract-specific quantification*. Neuroimage,39*(1), 336–47. doi:10.1016/j.neuroimage.2007.07.053.
